# Supplementary material for: Lipid-mediated gating of a miniature mechanosensitive MscS channel from Trypanosoma cruzi
Source: Nat Commun. 2025 Aug 8;16:7339. doi: 10.1038/s41467-025-62757-z (PMC12334622; doi:10.1038/s41467-025-62757-z)
Supplement: Supplementary file 1 — Supplementary Information [file 41467_2025_62757_MOESM1_ESM.pdf]

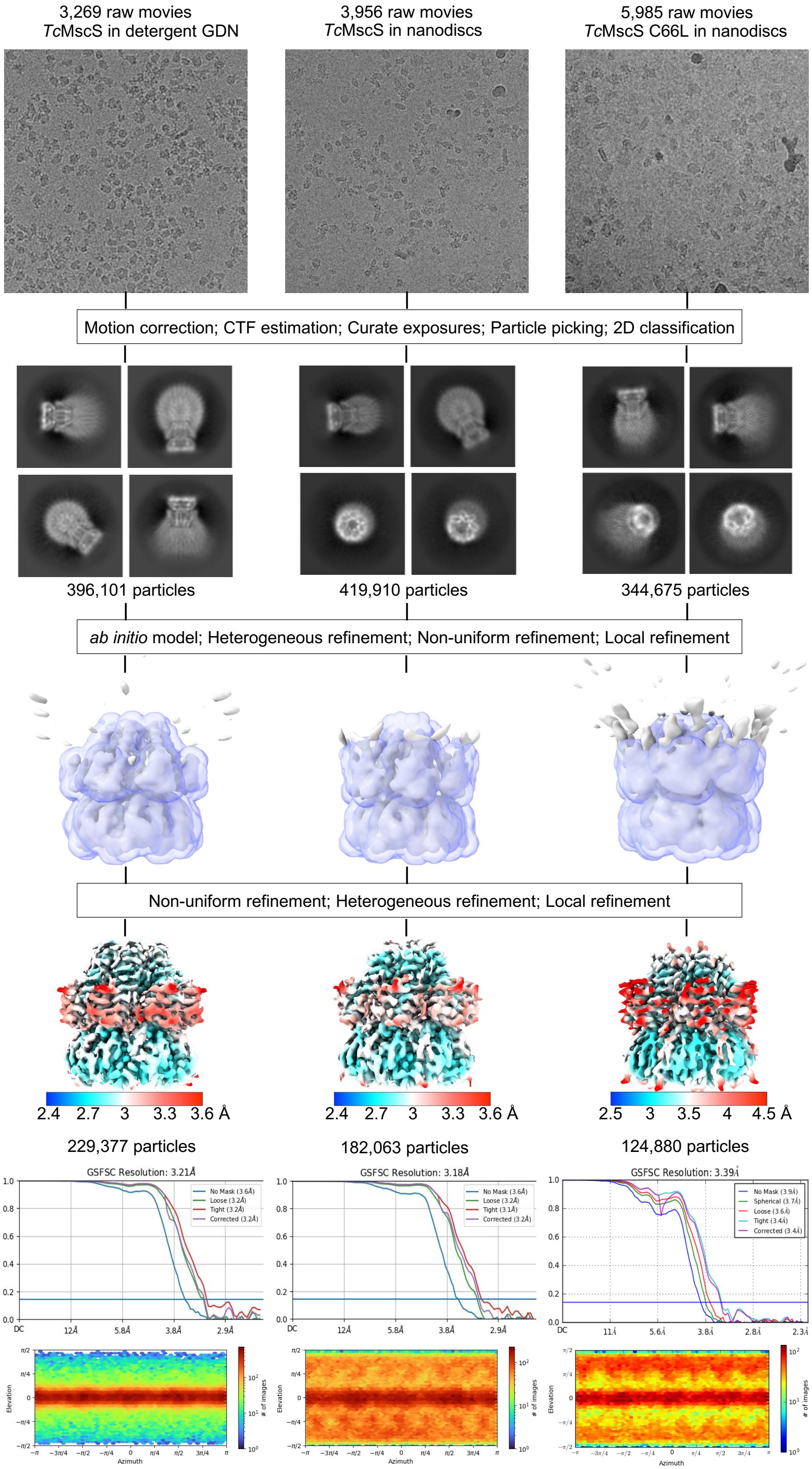

Supplementary Fig. 1 | Cryo-EM data processing and validation. Flow chart of data processing of the wild-type *Tc*MscS in detergents and in nanodiscs and the C66L mutant in nanodiscs. Fourier shell correlation (FSC) curves and final particle orientation distributions are also shown.

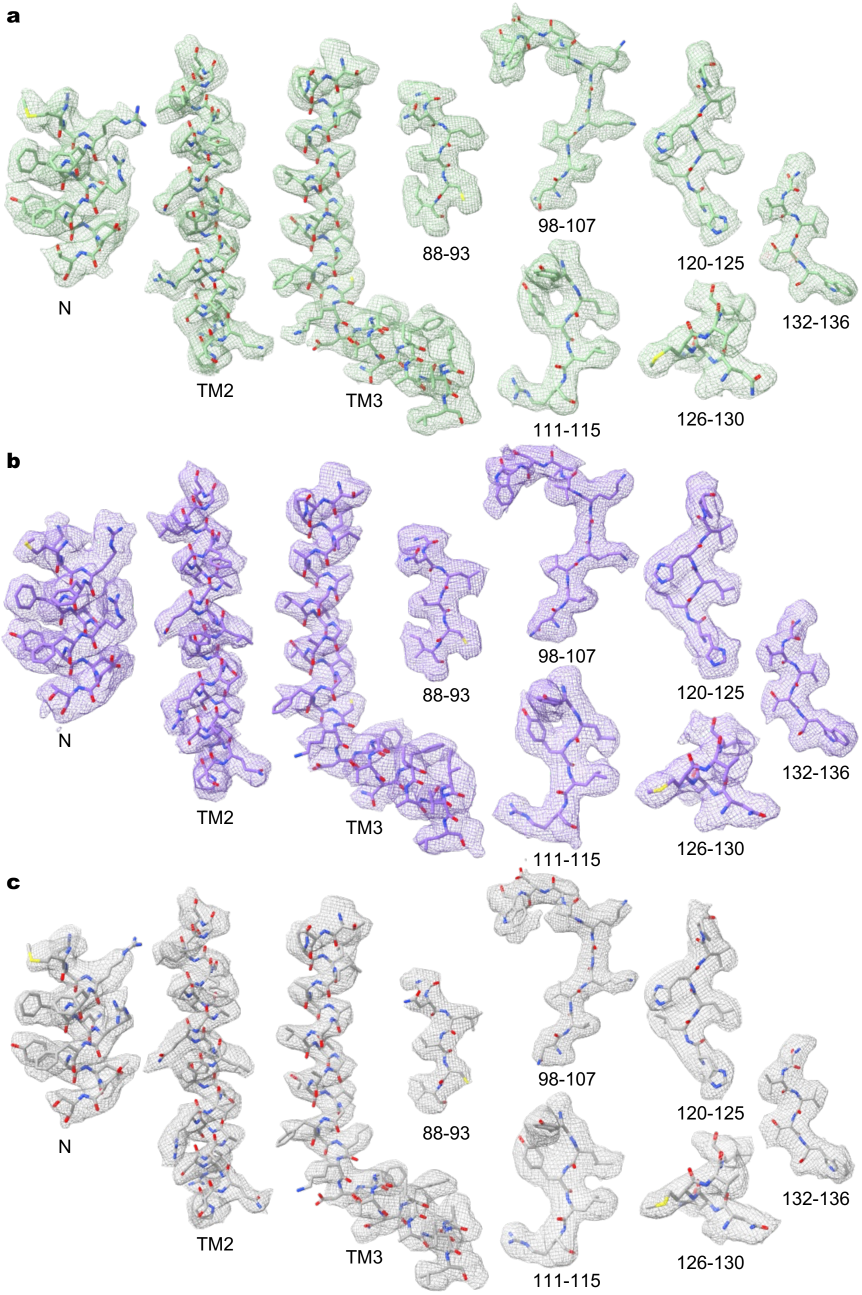

Supplementary Fig. 2 | Cryo-EM density. Segmented densities are shown for the wild-type *Tc*MscS in detergents (**a**) and in nanodiscs (**b**) and the C66L mutant in nanodiscs (**c**).

```
                                                                             N1            TM2
                                                                              10            20
TcMscS ---------------------------------------------------------MKRFFNRFYLDTGIIADPSQRS 22
EcMscS MEDLNVVDSINGAGSWLVANQALLLSYAVNIVAALAIIIVGLIIARMISNAVNRLMISR--KIDATVAD 67
VcMscS MEHLS------IAVDFLLTHKLLL----------TALILLGVISVRRL--VLEHIRGDAALITEKQ-RK 50

                    TM2                         TM3a                 TM3b             β1
          30        40        50        60        70        80        90
TcMscS LASRVSAFLVQGAVAFSLLGTIGVDTSPLIAAAGVTGATIGFACKDFGTNFVASIVLSGQQSIRTGNLV 91
EcMscS FLSALVRYGIIAFTLIAALGRVGVQTASVIAVLGAAGLAVGLALQGSLSNLAAGVLLVMFRPFRAGEYV 136
VcMscS WMS-RTKNGVFTVTLLALFMLWQSEISEFALSVTAIAVAIVVASKEIILCFTGSIQRASSRSFRIGDWI 118

                β2          β3                 β4     α1     β5        Unresolved
          100       110                 120       130             140
TcMscS CIGTGLNVVKGKVVDWDTRYLYLRSSE---------GHLLHVPNNMVLNSVVTWE---QEKKQNP-H-- 145
EcMscS DLGG----VAGTVLSVQIFSTTMRTAD---------GKIIVIPNGKIIAGNIINFS--REPVRRNEFII 190
VcMscS EVGS----VCGEVIEHNLMATVIQEIDLHHGQYNYTGKTATLPNSMFFSHPVKNLNFMKRYVFHNFKIV 183

                Unresolved
          150                           160
TcMscS ----ETDLP--KQDVVK----------AP--GDNAAKQ------------------------------ 165
EcMscS GVAYDSDIDQVKQILTNII--------QS--EDRILKDREMTV-----------RLNELGASSINFVVR 238
VcMscS VPEFVNLFPLV-PILHERIEGHFAHFMEVARRYNTVIERHAGVDLPSAEPHIEISSSSTGEQVVHV--M 249

TcMscS ------------------------------------------------------ 165
EcMscS VWSNSG---DLQN-VYWDVLERIKREFDAAG--ISFPYPQMDVNFKRVKEDKAA 286
VcMscS IFCPTERANQLEQLIRRDFMQEYERVFPRSTPTPIVPVPSAS------------ 291
```

Supplementary Fig. 3 | Sequence alignment. *Tc*MscS (GenBank: KAF8291389.1) sequence is aligned with *Ec*MscS (NCBI: NP_417399.1) and *Vc*MscS (NCBI: WP_000400692.1). Secondary structure elements are indicated accordingly. Critical residues discussed in the main text are highlighted.

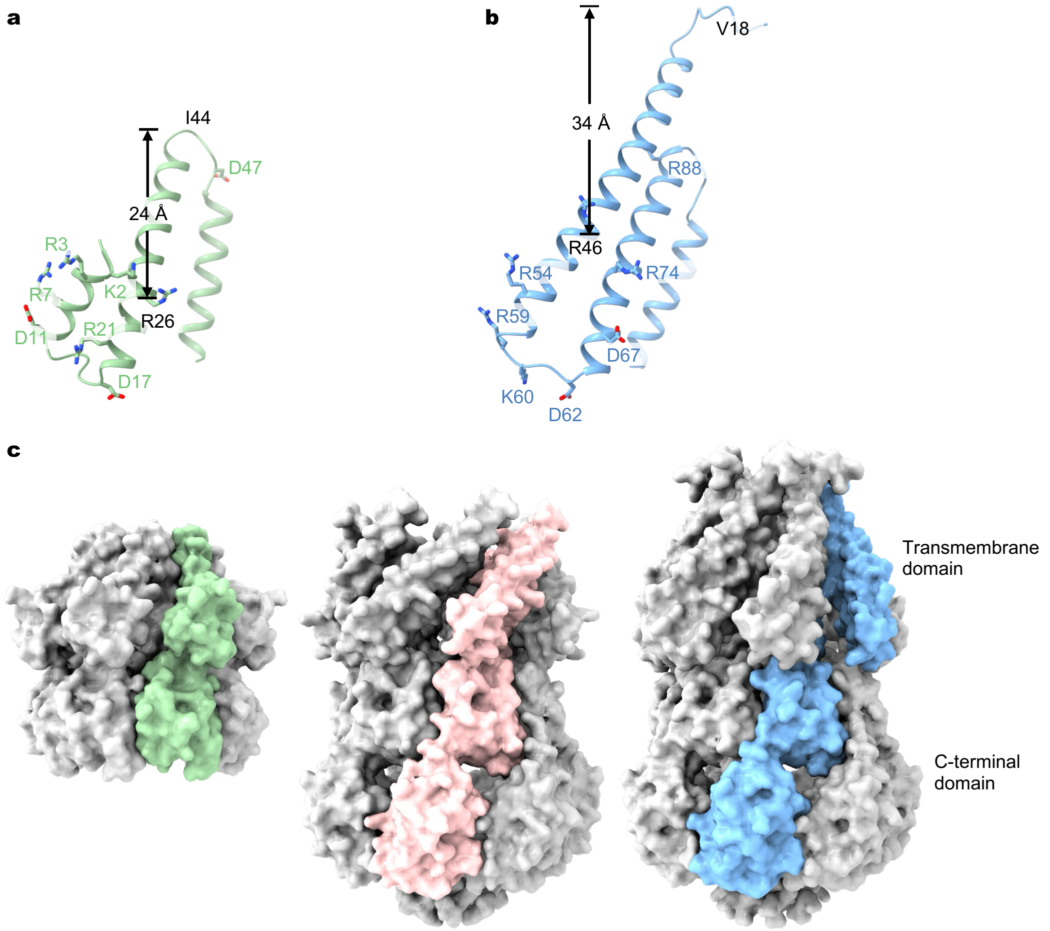

Supplementary Fig. 4 | Structural comparison of *Tc*MscS and *Ec*MscS. a-b, Estimation of the thickness of the transmembrane domain in *Tc*MscS (**a**) and *Ec*MscS (**b**, PDB: 6RLD). Charged residues are highlighted in sticks. **c**, Domain organizations of *Tc*MscS (left panel, non-domain swapped) and the open (middle panel, non-domain swapped) and closed *Ec*MscS channel (right panel, domain swapped). A single subunit of each heptameric channel is uniquely colored to illustrate the overall arrangements.

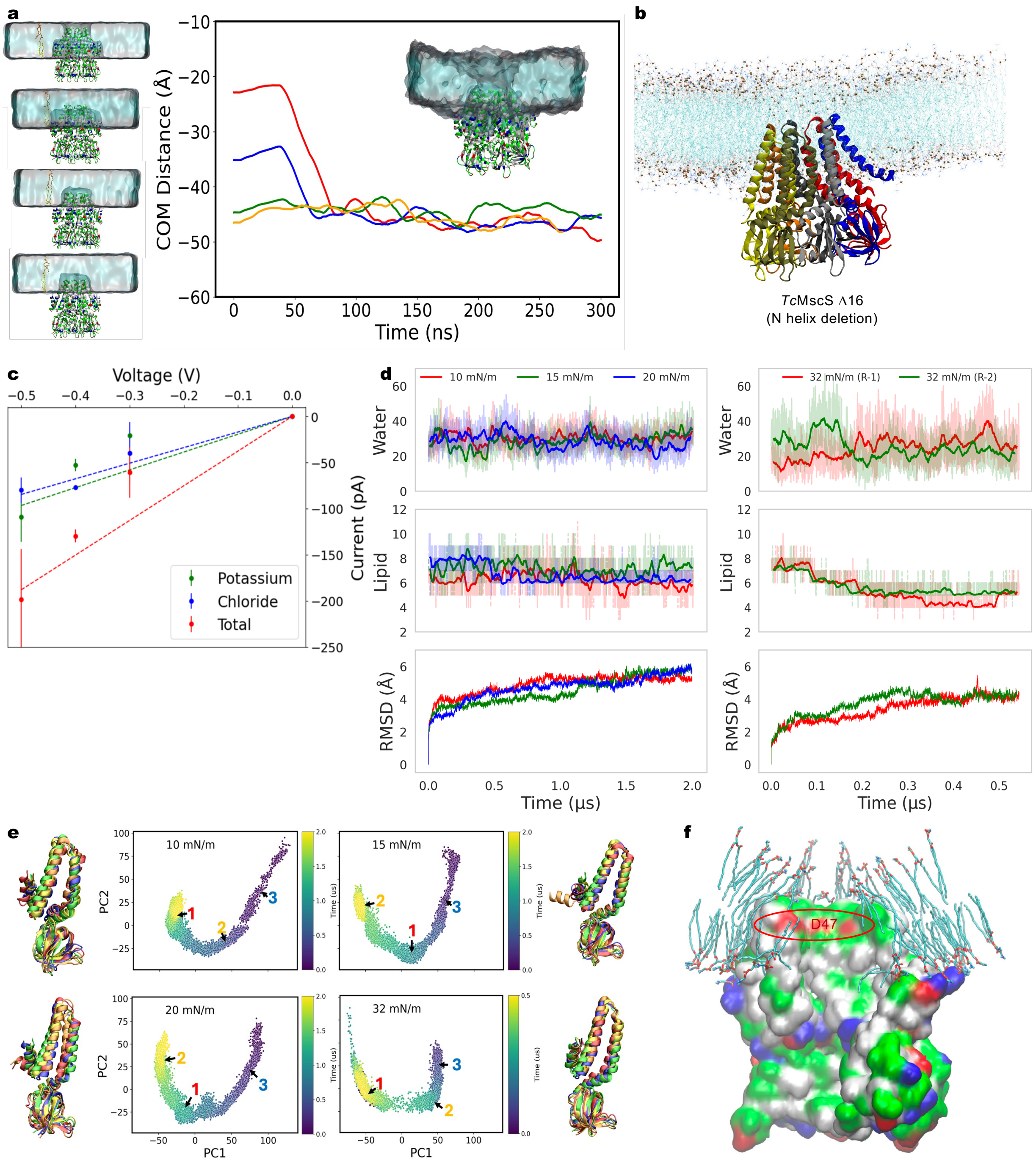

Supplementary Fig. 5 | MD simulations of *Tc*MscS. **a**, *Tc*MscS displacement along the z-axis in the POPC membrane. The center-of-mass distances between the protein and the lipid headgroups over the simulated time are plotted. The channel protein is colored by its residue type (white hydrophobic, blue positive, red negative, and green polar amino acids). **b**, Deletion of the entire N helix (Δ16) destabilizes channel partition in the membrane and loosens the TM2-TM3 packing. **c**, The I-V curves of the fully hydrated *Tc*MscS channel from all-atom MD simulations under applied potentials. Error bars represent the standard deviations from 2-3 replicas of 50 ns simulations. **d**, The time evolution of the number of water molecules, lipid tails inside the pore, and protein backbone RMSD with applied membrane tension in all-atom simulations. A cylindrical region (h=25 Å) was defined between residues D47 and F46, with a radius of 12 Å, for counting the number of pore lipids and water molecules. **e**, Conformational changes were analyzed using PCA on each all-atom trajectory. K-means clustering of the pairwise RMSD matrix identified dominant states, with the frame closest to each centroid selected as a representative structure. These structures were aligned to the Cryo-EM structure to illustrate conformational diversity. Cluster 1 (red) is the most populated, followed by Cluster 2 (orange) and Cluster 3 (blue). The pre-tension simulation frame is

shown in yellow, and the Cryo-EM structure in green. **f**, The cut-through view of protein in surface mode with residues colored by residue types.

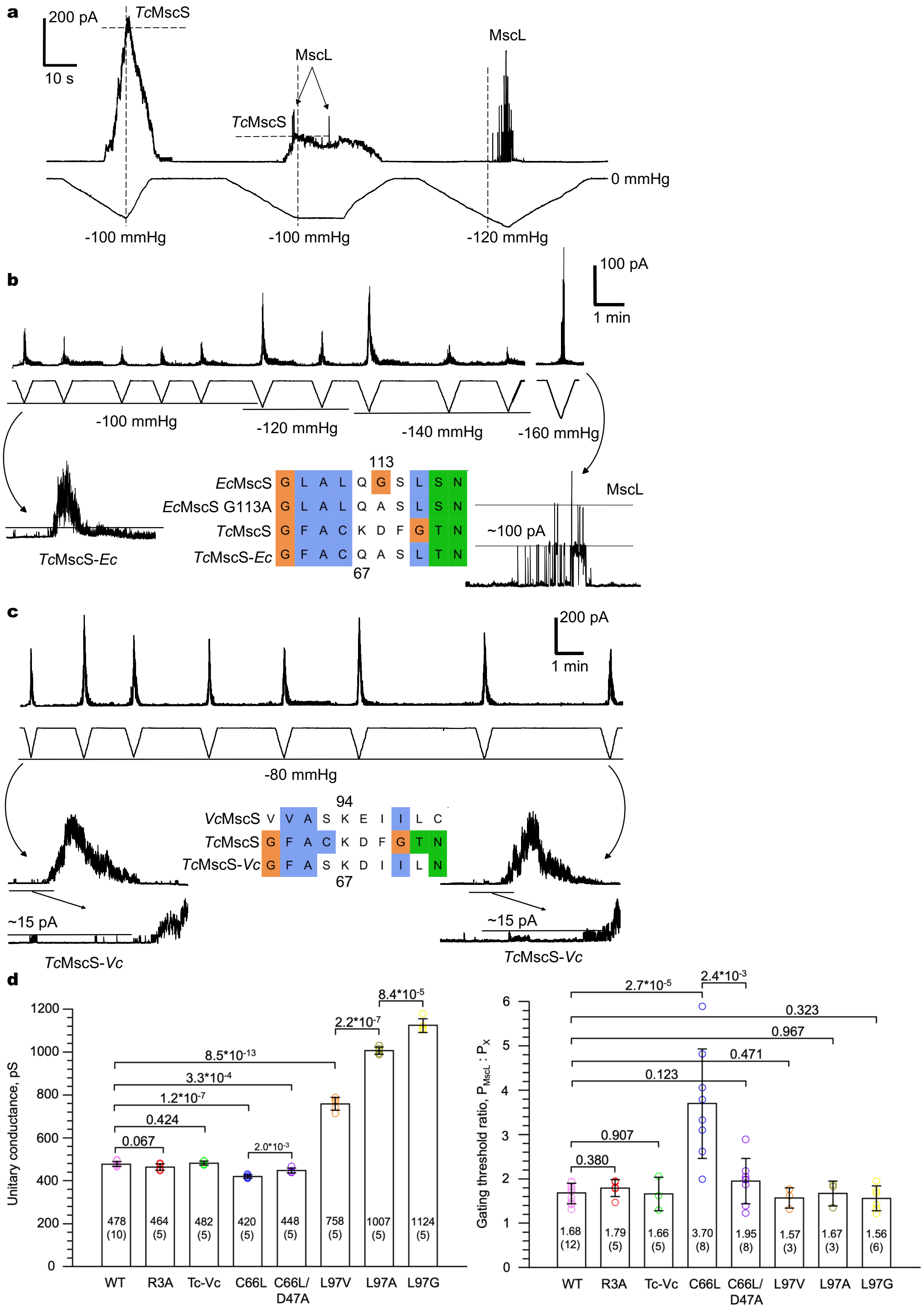

Supplementary Fig. 6 | Mutagenesis studies of *Tc*MscS. a, *Tc*MscS inactivation. The second application of pressure (-100 mmHg) resulted in much reduced *Tc*MscS currents with a few spikes of *Ec*MscL, while the third application of pressure (-120 mmHg) activated only *Ec*MscL channels. **b**, *Tc*MscS with a kink region from the non-inactivating *Ec*MscS-G113A mutant. **c**, *Tc*MscS with a kink region from the wild-type *Vc*MscS. Inactivation is still present in *Tc*MscS_to_*Ec*, but almost completely abolished in *Tc*MscS_to_*Vc*. **d**, Unitary conductance and gating pressure threshold of functional *Tc*MscS mutants (mean ± S.E.). The number of patches measured (in brackets) are indicated. P values are presented on top.

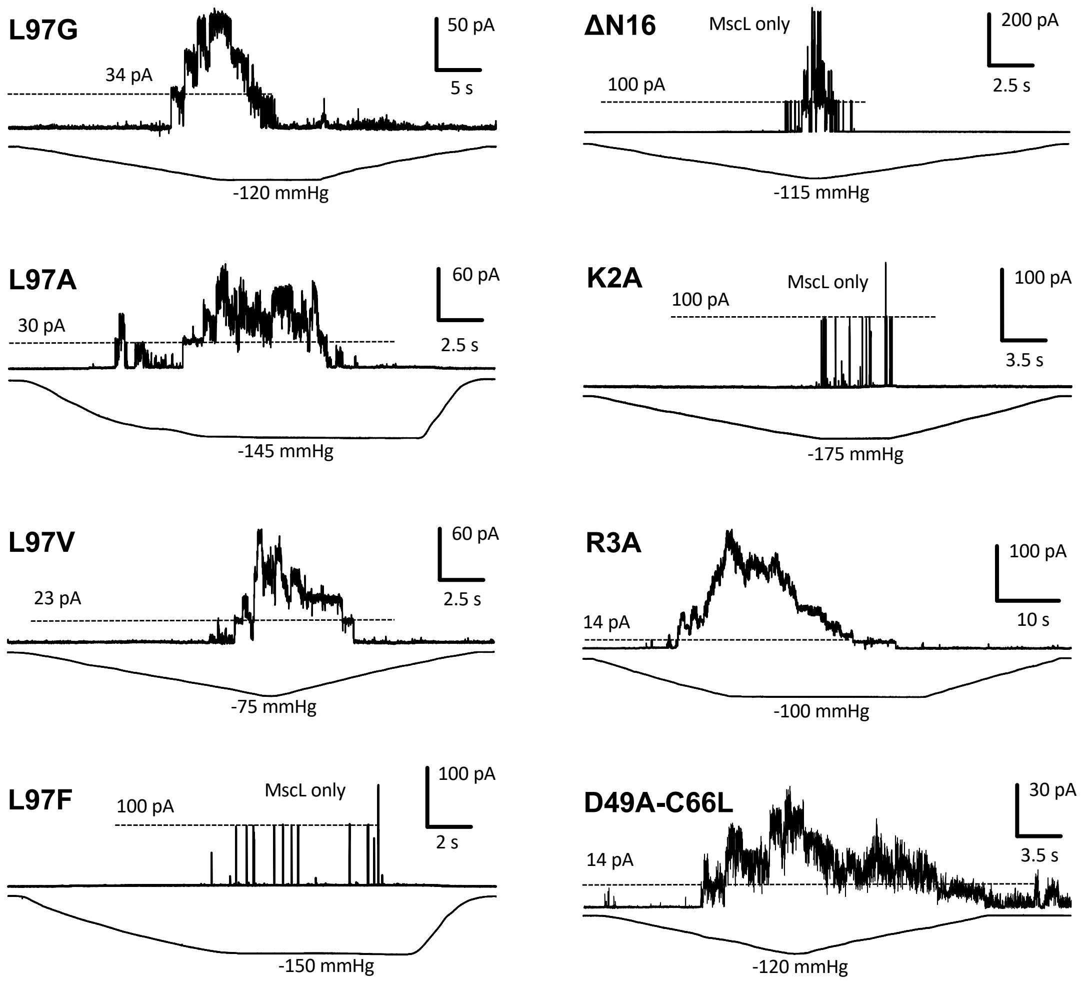

Supplementary Fig. 7 | Representative traces of activation of *Tc*MscS mutants by membrane tension. For the L97F, ΔN16, and K2A mutants, tension-activated currents were not observed. Only *Ec*MscL channels, used as an internal control, were activated by near-lytic tension in these excised membrane patches.

Supplementary Table 1 | Cryo-EM data collection, refinement and validation statistics

	*Tc*MscS in detergent (EMD-44520) (PDB 9BGQ)	*Tc*MscS in lipid nanodiscs (EMD-44521) (PDB 9BGS)	*Tc*MscS C66L in lipid nanodiscs (EMD-44522) (PDB 9BGT)
**Data collection and processing**			
Magnification	120k	120k	81k
Voltage (kV)	200	200	300
Electron exposure ($e^{-}/Å^2$)	43.11	42.46	54.33
Defocus range (µm)	-0.8 to -2.4	-0.8 to -2.4	-0.8 to -2.4
Pixel size (Å)	1.2	1.2	1.083
Symmetry imposed	C7	C7	C7
Initial particle images (no.)	2,193,852	1,467,083	2,244,568
Final particle images (no.)	229,377	182,063	124,880
Map resolution (Å)	3.2	3.2	3.4
FSC threshold	0.143	0.143	0.143
Map resolution range (Å)	2.4-3.6	2.4-3.6	2.5-4.5
**Refinement**			
Initial model used (PDB code)	AlphaFold	This study	This study
Model resolution (Å)	3.3	3.3	3.7
FSC threshold	0.5	0.5	0.5
Map sharpening *B* factor ($Å^2$)	-207.7	-197.1	-177.1
Model composition			
Nonhydrogen atoms	7,231	7,203	7,196
Protein residues	959	959	959
Ligands	0	0	0
B factors ($Å^2$)			
Protein	59.91	65.60	60.45
Ligand	N/A	N/A	N/A
R.m.s. deviations			
Bond lengths (Å)	0.002	0.002	0.002
Bond angles (°)	0.387	0.355	0.407
Validation			
MolProbity score	1.20	1.27	1.10
Clash score	4.11	5.03	3.10
Poor rotamers (%)	0	0	0
Ramachandran plot			
Favored (%)	98.52	99.26	99.26
Allowed (%)	1.48	0.74	0.74
Disallowed (%)	0	0	0

Supplementary Table 2 | MD simulation system details

System	Membrane System (xyz dimension in Å)	Number of atoms	Number of lipids (upper/lower)	Dynamics (3 x Replica)
**1**	POPC (215.21 x 209.33 x 193.86)	Total Atoms = 728915<br>Water = 189027<br>Protein = 14707<br>K^+ = 523, Cl^- = 544	POPC (U/L) = 564/526	350ns
**2**	POPC (169.69 x 170.28 x 185.06)	Total Atoms = 442326<br>Protein = 14,707<br>Water = 111106<br>K^+ = 307, Cl^- = 328	POPC (U/L) = 375/323	400ns
**3**	POPC (169.36 x 169.59 x 197.39)	Total Atoms = 472703<br>Protein = 14,707<br>Water = 119965<br>K^+ = 331, Cl^- = 396	POPC (U/L) = 375/352	250ns
**4**	POPC (170.51 x 170.69 x 195.51)	Total Atoms = 469317<br>Protein = 14707<br>Water = 119107<br>K^+ =327, Cl^- = 348	POPC (U/L) = 375/346	200ns
**5**	POPC (169.50 x 169.50 x 190.60)	Total Atoms = 457660<br>Protein = 14,588<br>Water = 116027<br>K^+ = 317, Cl^- = 337	POPC (U/L) = 375/329	2.5 μs
**6**	DLPC (208.64 x 209.38 x 193.41)	Total Atoms = 720045<br>Protein = 14707<br>Water = 193444<br>K^+ = 549, Cl^- = 570	DLPC (U/L) = 600/560	200ns
**7**	DLPC (169.50 x 169.50 x 185.32)	Total Atoms = 436916<br>Protein = 14707<br>Water = 114112<br>K^+ = 324 Cl^- = 345	DLPC (U/L) = 400/345	200ns

**8**	DLPC + DLPA (20%)<br>(170.51 x 170.14 x 172.12)	Total Atoms = 407561<br>Water = 105070<br>Protein = 14707<br>K^+ = 428, Cl^- = 299	DLPC (U/L) = 312/288<br>DLPA (U/L) = 78/72	200ns
**9**	DLPC + DLPA (20%)<br>(170.53 x 169.35 x 185.35)	Total Atoms = 437325<br>Water = 114500<br>Protein = 14707<br>K^+ = 457, Cl^- = 325	DLPC (U/L) = 436/329<br>DLPA (U/L) = 82/71	250ns
**10**	**(**D47A)<br>POPC<br>(169.69 x 170.28 x 185.06)	Total Atoms = 443564<br>Water = 111521<br>Protein = 14693<br>K^+ = 307, Cl^- = 335	POPC (U/L) = 392/324	100 ns (4 replicas)

POPC - 1-palmitoyl-2-oleoylphosphatidylcholine, **DLPC** - 1,2-dilauroyl-sn-glycero-3-phosphocholine, **DLPA** - 1,2-Dilauroyl-sn-glycero-3-phosphate. 150 mM KCl concentration was used in all systems. Cryo-EM structure of *Tc*MscS in GDN was used to construct most of the systems, except that the structure in lipid nanodiscs was used for system 5.

Supplementary Table 3 | Primers used for cloning

**Construct**	**Primer**	**Sequence (5′-3′)**
*Tc*MscS dN16	forward	GTCAGTCATATGGATCCCTCGCAG
	reverse	GTCAGTGAATTCCTGCTTCGCGGCG
*Tc*MscSL97F	forward	TTCAACGTGGTCAAAGGCAAAGTG
	reverse	GCCGGTGCCGATGCAC
*Tc*MscS L97A	forward	GCGAACGTGGTCAAAGGCAAAGTG
	reverse	GCCGGTGCCGATGCAC
*Tc*MscS L97V	forward	CATCGGCACCGGCGTGAACGTGGTCAA
	reverse	TTGACCACGTTCACGCCGGTGCCGATG
*Tc*MscS L97G	forward	GCATCGGCACCGGCGGGAACGTGGTCAAAG
	reverse	CTTTGACCACGTTCCCGCCGGTGCCGATGC
*Tc*MscS to *Ec*MscS	forward	CCATCGGCTTCGCGTGCCAGGCCTCCCTCACCAACTT CGTGGCGT
	reverse	ACGCCACGAAGTTGGTGAGGGAGGCCTGGCACGCGA AGCCGATGG
*Tc*MscS to *Vc*MscS	forward	CGACCATCGGCTTCGCGTCCAAGGACATCATCCTCAA CTTCGTGGCGTCGAT
	reverse	ATCGACGCCACGAAGTTGAGGATGATGTCCTTGGACG CGAAGCCGATGGTCG
*Tc*MscS C66L (for functional studies)	forward	CCATCGGCTTCGCGTTAAAGGACTTCGGCACC
	reverse	GGTGCCGAAGTCCTTTAACGCGAAGCCGATGG
*Tc*MscS C66L (for structural experiment)	forward	TGAAGGACTTCGGCACC
	reverse	ACGCGAAGCCGATGGTC
*Tc*MscS C66L D47A	forward	CCATCGGCGTCGCTACCTCGCCCCT
	reverse	AGGGGCGAGGTAGCGACGCCGATGG

Supplementary Table 4 | Summary of pore lipids in wild-type (WT) *Tc*MscS and D47A during MD simulations

System	Replica	Time (ns)	Tension (mN/m)	Voltage (mV)	Lipid		Water Permeation
					Initial	Final (Avg ± SD)	
WT	R-1	2500	0	0	0	0	Yes
	R-2	2500	0	0	0	0	Yes
	R-3	2500	0	0	0	0	Yes
WT	R-1	100	0	0	7	7	No
	R-2	100	0	0	7	7	No
	R-3	100	0	0	7	7	No
WT	R-1	30	0	500	7	7	No
	R-2	30	0	500	7	7	No
	R-3	30	0	500	7	7	No
WT	R-1	2000	10	0	7	6.2 ± 0.9	No
	R-1	2000	15	0	7	7.1 ± 0.8	No
	R-1	2000	20	0	7	6.2 ± 0.5	No
	R-1	500	32	0	7	4.6 ± 0.6	No
	R-2	500	32	0	7	5.2 ± 0.5	No
D47A	R-1	100	20	0	10	9.1 ± 1.9	No
	R-2	100	20	0	9	9.8 ± 1.7	No

Supplementary Table 5 | MD simulation checklist

**Reliability and reproducibility checklist for molecular dynamics simulations**<br>***All boxes must be marked YES by acceptance unless an N/A option is available**	**Yes**	**N/A**	**Response (Please state where this information can be found in the text)**
**1. Convergence of simulations and analysis**			
1a. Is an evaluation presented in the text to show that the property being measured has equilibrated in the simulations (*e.g.* time-course analysis)?	☒		Time-series data of protein RMSD, number of pore water and lipid molecules are presented in Supplementary Fig. 5.
1b. Then, is it described in the text how simulations are split into equilibration and production runs and how much data were analyzed from production runs?	☒		Method section “All-atom molecular dynamics simulation”
1c. Are there at least 3 simulations per simulation condition with statistical analysis?	☒		Supplementary Table 2
1d. Is evidence provided in the text that the simulation results presented are independent of initial configuration?	☒		We used different initial configurations.
**2. Connection to experiments**			
2a. Are calculations provided that can connect to experiments (*e.g.* loss or gain in function from mutagenesis, binding assays, NMR chemical shifts, J-couplings, SAXS curves, interaction distances or FRET distances, structure factors, diffusion coefficients, bulk modulus and other mechanical properties, *etc*.)?	☒		Loss of function mutant D47A data
**3. Method choice**			
3a. Is it described in the text what force field and water model are used and why?	☒		CHARMM36 protein and lipid force field in conjunction with TIP3P water model have been extensively validated for studying membrane proteins.
3b. Do simulations contain membranes, membrane proteins, intrinsically disordered proteins, glycans, nucleic acids, polymers, or cryptic ligand binding?	☒	☐	Response not needed if **N/A**
If 3b is **YES**, are enhanced sampling methods used?	☐	☒	Response not needed if **N/A**
If enhanced sampling methods are used, are the convergence criteria clearly stated?	☐		
If 3b is **YES**, is it explained in the text why or why not enhanced sampling methods are used?	☒		Membrane tension is applied to accelerate lipid or protein conformational changes.
**4. Code and reproducibility**			
4a. Is a table provided describing the system setup, such as simulation box dimensions, total number of atoms, total number of water molecules, salt concentration, lipid composition (number of molecules and type)?	☒		Supplementary Table 2
4b. Is it described in the text what simulation and analysis software and which versions are used?	☒		Method section “All-atom molecular dynamics simulation”
4c. Are initial coordinate and simulation input files and a coordinate file of the final output provided as supplementary files or in a public repository?	☒		https://github.com/LynaLuo-Lab/TcMscS
4d. Is there custom code or custom force field parameters?	☐	☒	Response not needed if **N/A**
If **YES**, are they provided as supplementary profiles or in a public repository?	☐		
