# Supplementary material for: Lipid-mediated gating of a miniature mechanosensitive MscS channel from Trypanosoma cruzi
Source: Nat Commun. 2025 Aug 8;16:7339. doi: 10.1038/s41467-025-62757-z (PMC12334622; doi:10.1038/s41467-025-62757-z)
Supplement: Supplementary file 2 — Description of Additional Supplementary Files [file 41467_2025_62757_MOESM2_ESM.pdf]

Description of Additional Supplementary Files

File name: Supplementary Movie 1
Description: Partial departure of pore lipids in all-atom MD simulation under lateral tension of 32 mN/m. The *Tc*MscS channel is shown in surface mode and colored by subunit. Lipids are shown in licorice mode (VMD nomenclature). Water oxygen atoms inside the pore are depicted as red dots. Lipid coordinates were smoothed over 10 frames (0.1 ns/frame) for clarity.
