# Supplementary material for: Lipid-mediated gating of a miniature mechanosensitive MscS channel from Trypanosoma cruzi
Source: Nat Commun. 2025 Aug 8;16:7339. doi: 10.1038/s41467-025-62757-z (PMC12334622; doi:10.1038/s41467-025-62757-z)
Supplement: Supplementary file 4 — Reporting Summary [file 41467_2025_62757_MOESM4_ESM.pdf]

nature portfolio

Corresponding author(s): Yun Lyna Luo, Peng Yuan

Last updated by author(s): Jul 11, 2025

Reporting Summary

Nature Portfolio wishes to improve the reproducibility of the work that we publish. This form provides structure for consistency and transparency in reporting. For further information on Nature Portfolio policies, see our Editorial Policies and the Editorial Policy Checklist.

Statistics

For all statistical analyses, confirm that the following items are present in the figure legend, table legend, main text, or Methods section.

n/a	Confirmed	
☐	☒	The exact sample size (*n*) for each experimental group/condition, given as a discrete number and unit of measurement
☐	☒	A statement on whether measurements were taken from distinct samples or whether the same sample was measured repeatedly
☒	☐	The statistical test(s) used AND whether they are one- or two-sided<br>*Only common tests should be described solely by name; describe more complex techniques in the Methods section.*
☒	☐	A description of all covariates tested
☒	☐	A description of any assumptions or corrections, such as tests of normality and adjustment for multiple comparisons
☐	☒	A full description of the statistical parameters including central tendency (e.g. means) or other basic estimates (e.g. regression coefficient) AND variation (e.g. standard deviation) or associated estimates of uncertainty (e.g. confidence intervals)
☒	☐	For null hypothesis testing, the test statistic (e.g. *F*, *t*, *r*) with confidence intervals, effect sizes, degrees of freedom and *P* value noted<br>*Give P values as exact values whenever suitable.*
☒	☐	For Bayesian analysis, information on the choice of priors and Markov chain Monte Carlo settings
☒	☐	For hierarchical and complex designs, identification of the appropriate level for tests and full reporting of outcomes
☒	☐	Estimates of effect sizes (e.g. Cohen's *d*, Pearson's *r*), indicating how they were calculated

Our web collection on statistics for biologists contains articles on many of the points above.

Software and code

Policy information about availability of computer code

Data collection	Cryo-EM: EPU 2 (ThermoFisher Scientific), leginon 3.7; Electrophysiology: pCLAMP 8.2 (Molecular Devices); Molecular dynamics: AMBER2020, Gromacs 2024.2.
Data analysis	Cryo-EM: cryoSPARC V3.4.0, V3.3.1 and V2.15.0, COOT v0.9.6, HOLE 2.2.005, PHENIX 1.20.1, MolProbity 4.4, UCSF CHIMERA 1.14 , UCSF CHIMERAX 1.5; Electrophysiology: ClampFit 10.6 (Molecular Devices); General data analysis: QtiPlot 1.1.5; Molecular dynamics: VMD19.3, MOSAICS, MDAnalysis python package

For manuscripts utilizing custom algorithms or software that are central to the research but not yet described in published literature, software must be made available to editors and reviewers. We strongly encourage code deposition in a community repository (e.g. GitHub). See the Nature Portfolio guidelines for submitting code & software for further information.

Data

Policy information about availability of data

All manuscripts must include a data availability statement. This statement should provide the following information, where applicable:

- Accession codes, unique identifiers, or web links for publicly available datasets
- A description of any restrictions on data availability
- For clinical datasets or third party data, please ensure that the statement adheres to our policy

The cryo-EM maps and atomic coordinates have been deposited to the Electron Microscopy Data Bank (accession codes: EMD-44520 [https://www.ebi.ac.uk/emdb/

EMD-44520], EMD-44521 [https://www.ebi.ac.uk/emdb/EMD-44521], and EMD-44522 [https://www.ebi.ac.uk/emdb/EMD-44522]) and Protein Data Bank (PDB entry codes: 9BGQ [https://www.rcsb.org/structure/9BGQ], 9BGS [https://www.rcsb.org/structure/9BGS], 9BGT [https://www.rcsb.org/structure/9BGT]). The raw images have been deposited to the Electron Microscopy Public Image Archive (accession codes: EMPIAR-12056 [https://www.ebi.ac.uk/empiar/EMPIAR-12056], EMPIAR-12057 [https://www.ebi.ac.uk/empiar/EMPIAR-12057], and EMPIAR-12058 [https://www.ebi.ac.uk/empiar/EMPIAR-12058]). For molecular dynamics simulation data, initial coordinates, simulation input files, and a coordinate file of the final output are available at https://github.com/LynaLuo-Lab/TcMscS. All numerical data related to Supplementary Fig. 6d are provided as a Source Data file with this paper.

Research involving human participants, their data, or biological material

Policy information about studies with human participants or human data. See also policy information about sex, gender (identity/presentation), and sexual orientation and race, ethnicity and racism.

Reporting on sex and gender	N/A
Reporting on race, ethnicity, or other socially relevant groupings	N/A
Population characteristics	N/A
Recruitment	N/A
Ethics oversight	N/A

Note that full information on the approval of the study protocol must also be provided in the manuscript.

Field-specific reporting

Please select the one below that is the best fit for your research. If you are not sure, read the appropriate sections before making your selection.

☒ Life sciences ☐ Behavioural & social sciences ☐ Ecological, evolutionary & environmental sciences

For a reference copy of the document with all sections, see nature.com/documents/nr-reporting-summary-flat.pdf

Life sciences study design

All studies must disclose on these points even when the disclosure is negative.

Sample size	Electrophysiology:<br>Experiments were repeated sufficiently to ensure reproducibility, N =5 or more for a single data point, reflected in error bars in the data.<br>Cryo-EM: 3269, 3956 and 5985 movies were collected for the wild-type TcMscS in detergent, in nanodiscs, and TcMscS C66L in nanodiscs. This sample size is sufficient for 3D reconstruction.
Data exclusions	Electrophysiology:<br>No data were excluded from presentation.<br>Cryo-EM: 3226, 3584 and 5586 good images for the wild-type TcMscS in detergent, in nanodiscs, and TcMscS C66L in nanodiscs were manually selected for blob picking and template picking.
Replication	Experiments were repeated sufficiently to ensure reproducibility, every patch was excised from a different cell. All attempts at replication were successful.
Randomization	Experiments were randomized with respect to constructs being patch-clamped: cells expressing channels were randomly selected.
Blinding	The investigators were blinded to group allocation during data collection and analysis.

Reporting for specific materials, systems and methods

We require information from authors about some types of materials, experimental systems and methods used in many studies. Here, indicate whether each material, system or method listed is relevant to your study. If you are not sure if a list item applies to your research, read the appropriate section before selecting a response.

Materials & experimental systems

n/a	Involved in the study
☒	☐ Antibodies
☐	☒ Eukaryotic cell lines
☒	☐ Palaeontology and archaeology
☒	☐ Animals and other organisms
☒	☐ Clinical data
☒	☐ Dual use research of concern
☒	☐ Plants

Methods

n/a	Involved in the study
☒	☐ ChIP-seq
☒	☐ Flow cytometry
☒	☐ MRI-based neuroimaging

Eukaryotic cell lines

Policy information about cell lines and Sex and Gender in Research

Cell line source(s)	Pichia pastoris SMD1163H.
Authentication	None of the cell line used were authenticated.
Mycoplasma contamination	The cell lines were not tested for mycoplasma contamination.
Commonly misidentified lines (See ICLAC register)	N/A

Plants

Seed stocks	N/A
Novel plant genotypes	N/A
Authentication	N/A
